# Effects of obesity on the serum BMP15, GDF9, and kisspeptin concentrations in women of reproductive age

**DOI:** 10.5937/jomb0-37329

**Published:** 2023-08-25

**Authors:** Funda Bulut Arıkan, Nevin Sagsoz

**Affiliations:** 1 Kirikkale University, Faculty of Medicine, Department of Physiology, Kirikkale, Turkey; 2 Kirikkale University, Faculty of Medicine, Department of Obstetrics and Gynecology, Kirikkale, Turkey

**Keywords:** obesity, BMP15, GDF9, kisspeptin, reproductive, gojaznost, BMP15, GDF9, kispeptin, reproduktivni

## Abstract

**Background:**

As BMP15, GDF9, and kisspeptin all play critical roles in folliculogenesis and fertilization, investigating the possible relationship between obesity and these three factors could prove crucial in relation to understanding the role of obesity in infertility. Thus, the present study sought to determine the effects of obesity on the serum BMP15, GDF9, and kisspeptin concentrations in women of reproductive age.

**Methods:**

Ninety female participants were equally divided into three groups: class-1 obese (n=30), class-2 obese (n=30), and normal weight (control; n=30). The participants' serum BMP15, GDF9, and AMH concentrations were measured. Moreover, the serum kisspeptin concentrations were evaluated in the class-1 obese and control groups by means of the enzyme-linked immunosorbent assay (ELISA) method while the participants were in their menstrual period.

**Results:**

The serum BMP15 and kisspeptin concentrations were found to be much higher in the control group than in both obese groups (p=0.001 and p=0.01, respectively). While the GDF9 concentration exhibited a statistically significant positive correlation with age, the BMP15 concentration exhibited a positive correlation with the kisspeptin and LH concentrations in the control group. In addition, a positive correlation was identified between the BMP15 concentration and both age and the glucose level and a negative correlation with the insulin level in both the obese groups.

**Conclusions:**

Obesity appears to reduce the serum BMP15 and kisspeptin concentrations in obese women of reproductive age. This reduction may represent a milestone in reproductive dysfunction and may be used to predict the success of infertility treatment in obese women.

## Abstract (sr)

**Uvod:**

Kako BMP15, GDF9 i kispeptin igraju kritičnu ulogu u folikulogenezi i oplodnji, istraživanje moguće veze između gojaznosti i ova tri faktora bi se moglo pokazati ključnim u odnosu na razumevanje uloge gojaznosti u neplodnosti. Stoga je ova studija nastojala da utvrdi efekte gojaznosti na koncentracije BMP15, GDF9 i kispeptina u serumu kod žena u reproduktivnom dobu.

**Metode:**

Devedeset učesnica je podeljeno u tri grupe: gojaznost klasa 1 (n=30), gojaznost klasa 2 (n=30) i normalna težine (kontrolni subjekti; n=30). Izmerene su koncentracije BMP15, GDF9, i AMH u serumu učesnika. Štaviše, koncentracija kispeptina u serumu je procenjena u grupi gojaznih klase 1 i u kontrolnoj grupi metodom enzimskog imunosorbentnog testa (ELISA) dok su učesnice bile u menstrualnom periodu.

**Rezultati:**

Utvrđeno je da su koncentracije BMP15 i kaspentina u serumu mnogo veće u kontrolnoj grupi nego u obe gojazne grupe (p=0,001 i p=0,01, respektivno). Dok je koncentracija GDF9 pokazala statistički značajnu pozitivnu korelaciju sa godinama, koncentracija BMP15 je pokazala pozitivnu korelaciju sa koncentracijama kispeptina i LH u kontrolnoj grupi. Pored toga, identifikovana je pozitivna korelacija između koncentracije BMP15 i starosti i nivoa glukoze i negativna korelacija sa nivoom insulina u obe gojazne grupe.

**Zaključak:**

Čini se da gojaznost smanjuje serumske koncentracije BMP15 i kispeptina kod gojaznih žena u reproduktivnom dobu. Ovo smanjenje može da predstavlja prekretnicu u reproduktivnoj disfunkciji i može da se koristi za predviđanje uspeha lečenja.

## Introduction

Obesity is a severe disorder that lowers the basal metabolic rate and increases the chance of metabolic diseases such as diabetes mellitus [1] [2] [3]. Furthermore, obesity is very common in women of reproductive age [4]. Obesity has been reported to have an adverse effect on reproductivity and is associated with many complications such as infertility, ovulation abnormalities, impaired follicle growth and development, hormonal imbalance, oocyte mitochondrial dysfunction, and menstrual dysfunction due to an impaired ovarian axis [4] [5] [6] [7].

As the body mass index (BMI) increases, the rate of pregnancy in eumenorrheic women and in vitro fertilization (IVF) success rates may decrease [8] [9]. Furthermore, obesity is related to decreased estradiol levels, reduced number of healthy fertilized oocytes, and lower fecundability and live birth rates [7].

Growth and differentiation factor-9 (GDF9) and bone morphogenetic protein-15 (BMP15) are transforming growth factor beta (TGF-β) superfamily members that are expressed in oocytes, especially during folliculogenesis [10]. GDF9 and BMP15 play crucial roles in the control of female reproduction by regulating folliculogenesis and ovulation [2] [3]. These factors also regulate the oocyte maturation and proliferation and the differentiation of granulosa cells [10] [11].

Another TGF-β superfamily member, the anti-Müllerian hormone (AMH) is expressed by the granulosa cells in the gonads and is also involved in the regulation of folliculogenesis. AMH serum concentrations accurately indicate the size of the antral follicle pool representing the remaining number of primordial follicles [12]. BMP-15 and GDF-9 have been reported to be related to menopausal age, and ovarian failure [13], suggesting a relationship between these two factors and AMH is plausible.

Another peptide that has been investigated is kisspeptin. Kisspeptin is a potent neuropeptide that is synthesized by the neurons of the rostral periventricular area of the third ventricle (RP3V) and the arcuate nucleus in the hypothalamus [14]. Kisspeptin neurons stimulate gonadotropin-releasing hormone (GnRH) neurons and induce follicle-stimulating hormone (FSH) and luteinizing hormone (LH) secretion. Therefore, these neurons play a crucial role in fertilization [14] [15]. As well as the regulation role in fertilization of kisspeptin, it was also reported to have severe effects on metabolism, obesity, and glucose homeostasis [16].

Considering the impact of obesity, BMP15, GDF-9 and kisspeptin on folliculogenesis, and fertilization, investigating the possible relationship between obesity and these three factors could prove crucial in relation to understanding the role of obesity in infertility.

## Materials and methods

### Subjects

Required approvals were obtained from Kirikkale University clinical research ethics committee for the study. Before participating, all volunteers were informed in detail about the study and informed consent was received from each volunteer. This study was conducted in accordance with the Declaration of Helsinki.

This study consisted of 90 female volunteers who were divided evenly into three groups - class-1 obese, class-2 obese, and normal weight (control) for serum GDF9, BMP15 and AMH analysis. Serum kisspeptin levels were evaluated as a preliminary study in the class-1 obese and normal-weight female volunteers. Care was taken to ensure that the mean age of the volunteers was similar. All participants had regular menstrual patterns (24-38 days/cycle) in the reproductive period and registered at the Gyna ecology and obstetrics clinic of Kirikkale University Medical Faculty Hospital.

Exclusion criteria: having diseases such as cancer, tuberculosis, hepatitis, AIDS, endocrine diseases (such as diabetes and thyroid), cardiac and renal diseases, smoking, used or using hormone drugs (oral contraceptives and progesterone) in the last year.

Inclusion criteria: having regular menstruation pattern and not having exclusion criteria.

### Study protocol

Participants’ menstrual patterns and demographic characteristics, such as age, weight, height, etc., were recorded. BMI (weight (kg)/height (m)^2^) were calculated. BMI was categorized as normal (BMI 18.5–24.9), class-1 obese (BMI 30.0–34.9), and class-2 obese (BMI 35.0–39.9).

After eight hours of fasting, venous blood samples were drawn from a forearm vein during the menstrual period (Days 2–3) in the morning. All blood samples were centrifuged to obtain serum within 30 minutes after bloodletting. Serum samples were then kept at -80°C until GDF-9, BMP-15, kisspeptin, AMH, FSH, LH, estradiol, glucose, and insulin serum level analysis were performed. The homeostatic model assessment of insulin resistance index (HOMAIR) was utilized to quantify insulin resistance = Fasting glucose (mmol/L) x Fasting serum insulin (μU)/L)/ 405.

Serum insulin, glucose, FSH, LH, and estradiol levels were measured using the electrochemiluminescence immunoassay technique with the Cobas 8000 analyzer using Roche Cobas kits (Roche Diagnostics GmbH, Mannheim, Germany). The quantitative ELISA kits protocol used to measure the main peptides are shown in Table 1.

**Table 1:** Quantitative ELISA kits information.

	Catalogue<br>Number	Detection<br>Range	Intra-assay (IA)<br>and Inter-assay (IA)<br>Precision	Microplate<br>Reader	Sensitivity
GDF9	MyBioSource Inc.<br>(San Diego, USA).<br>Catalogue Number:<br>MBS771756	501600<br>pg/mL	IA CV <10%IA<br>CV <15%	BioTek® ELx800<br>(BioTek<br>Instruments,Inc. Northern<br>Vermont,USA)	10 pg/mL
BMP15	MyBioSource Inc.<br>(San Diego, USA).<br>Catalogue Number:<br>MBS770624	20–1000<br>pg/mL	IA CV <10%IA<br>CV <15%	BioTek® ELx800<br>(BioTek Instruments,<br>Inc. Northern<br>Vermont,USA)	10 pg/mL
Kisspeptin	Phoenix Human<br>Kiss-1 ELISA kit<br>(Phoenix Pharmaceuticals.<br>Inc. Burlingame, CA, USA)<br>Catalogue Number:<br>EK-048-56	0–100<br>ng/mL	IA CV <10%IA<br>CV <15%	BioTek® ELx800<br>(BioTek<br>Instruments,Inc. Northern<br>Vermont, USA)	0.08 ng/mL
AMH	Elabscience Biotechnology<br>Inc. (Houston, USA)<br>Catalogue Number:<br>E-EL-H0317	93.75–6000<br>pg/mL	The coefficient<br>of variation is<br><10%.	BioTek® ELx800<br>(BioTek Instruments,<br>Inc. Northern<br>Vermont, USA)	56.25 pg/mL

### Statistical analysis

The statistical analyses were performed using SPSS, version 20.0 (IBM SPSS Statistics, Armonk, NY,USA), and the significance level was set at *p* < .05. Normality analysis was conducted with the Shapiro-Wilk test. Differences among the means of parameters in the obese and control groups were determined using a one-way ANOVA test.

The mean serum kisspeptin was compared between the class-1 obese and control groups through an independent sample t-test. The correlation analyses were done using Pearson or Spearman two-way correlation tests, determined by the normality of the data.

## Results

The demographic and laboratory parameters of the groups are shown in Table 2. The mean serum BMP15, glucose, insulin, and HOMA levels were statistically significant among the class-1 obese, class-2 obese, and control groups (p<0.05, Table 2).

**Table 2:** Demographic and laboratory parameters of the groups. *P-value was determined with a one-way ANOVA test between groups. (^*^p<0.05. ^**^p 0.005).

Parameters	Control<br>(Mean ± STD)	Obese 1 group<br>(Mean ± STD)	Obese 2 group<br>(Mean ± STD)	P value
Age	29.1 ± 7.7	31.9 ± 8.8	32.8 ±7.2	0.18
BMI (kg/m^2^)	22.4 ± 2.2	31.2 ± 0.8	36.4 ± 0.9	0.000^**^
FSH (mIU/L)	6.4±1.3	6.1 ±1.6	6.8±1.8	0.37
LH (mIU/L)	6.3±3.6	5.7 ±2.5	6.5±2.7	0.65
LH/FSH ratio	1.01±0.6	0.94 ± 0.5	1.1 ± 0.7	0.74
Estradiol (pmol/L)	159.2 ± 95.3	180.9 ± 107.8	173.5 ± 110.3	0.80
AMH (ng/L)	358.9 ± 82.8	378.7 ±188.6	376.2±162.2	0.87
GDF9 (ng/L)	202.9 ± 43.8	201.3 ± 50.7	203.4 ± 35.8	0.98
BMP15 (ng/L)	169.8 ±54.2	125.5 ±32.4	141.9 ±38.1	0.001^**^
Kisspeptin (μg/L)	1.39 ±0.16	1.14 ± 0.49		0.01^*^
Glucose (mmol/L)	4.9 ± 0.52	5.09 ±0.47	5.4 ±0.42	0.005^**^
Insulin ( mIU/L)	12.1±4.9	10.2±6	14.5±6.1	0.03^*^
HOMA	2.2±0.8	2.1±1.3	3.2±1.4	0.007

The mean HOMA, serum insulin, and glucose levels were highest in the class-2 obese group compared with the class-1 obese and control groups. The BMP15 serum concentrations were much higher in the control group than in the class-1 and class-2 obese groups (p = 0.001 for both). Similarly, the serum kisspeptin concentrations were determined to be higher in the control group than in the class-1 obese group (p = 0.01). The findings concerning both obese groups were compared with those concerning the control group using the Turkey posthoc HSD test, as shown in Table 3.

**Table 3:** Post-Hoc tests between groups. P-value was determined as obese groups were compared with the control group by Tukey HSD test.<br>P-value of the Tukey HSD test of insulin between obese 1 and obese 2 groups = 0.02<br>P-value of the Tukey HSD test of HOMA between obese1 and obese 2 groups = 0.012

Parameters	Obese 1 group<br>P value	Obese 2 group<br>P value
BMP15 (ng/L)	0.000^**^	0.03^*^
BMI (kg/m^2^)	0.000^**^	0.000^**^
Glucose (mmol/L)	0.40	0.004^**^
Insulin (mIU/L)	0.52	0.33
HOMA	0.98	0.02^*^

Correlation analyses were also performed, and the results are presented in Table 4.

**Table 4:** Correlations analyses.

Both Obese<br>Groups	GDF9<br>r	p	BMP15<br>r	p	Kisspeptin<br>r	p	BMI<br>r	p
Age	0.157	0.22	0.397	0.002^*^	-0.179	0.35	0.095	0.47
FSH	-0.047	0.78	0.279	0.10	-0.162	0.53	0.250	0.14
LH	-0.166	0.31	-0.072	0.66	-0.327	0.17	0.213	0.19
LH/FSH ratio	-0.071	0.68	-0.176	0.31	-0.352	0.18	0.128	0.46
Estradiol	0.054	0.74	0.222	0.18	-0.101	0.67	0.057	0.73
AMH	0.248	0.07	0.111	0.43	0.024	0.90	0.094	0.51
Glucose	-0.022	0.87	0.325	0.02^*^	-0.040	0.84	0.339	0.016^*^
Insulin	-0.150	0.29	-0.297	0.03^*^	0.076	0.73	0.387	0.007^**^
HOMA	-0.144	0.34	-0.259	0.09	0.039	0.86	0.429	0.004^**^
Triglyceride	0.052	0.76	-0.276	0.10	0.025	0.93	-0.059	0.73
LDL	-0.025	0.88	-0.016	0.92	-0.056	0.85	-0.165	0.33
HDL	0.050	0.77	0.165	0.33	0.059	0.83	-0.100	0.55
Total Cholesterol	-0.027	0.87	-0.057	0.74	-0.020	0.94	0.001	0.99
Kisspeptin	0.197	0.30	-0.213	0.77			-0.105	0.05
	GDF9		BMP15		Kisspeptin		BMI	
Control Group	r	p	r	p	r	p	r	p
Age	0.530	0.004^**^	0.342	0.07	0.262	0.17	0.170	0.38
FSH	0.061	0.78	0.184	0.41	0.076	0.73	-0.121	0.59
LH	0.313	0.16	0.442	0.04^*^	0.205	0.37	-0.213	0.35
LH/FSH ratio	0.344	0.12	0.384	0.08	-0.064	0.78	-0.149	0.52
Estradiol	0.244	0.28	0.339	0.13	0.088	0.70	-0.324	0.15
AMH	-0.033	0.87	0.019	0.92	0.025	0.90	-0.005	0.97
Glucose	0.018	0.93	0.067	0.76	-0.041	0.85	0.058	0.79
Insulin	-0.110	0.63	0.178	0.44	0.077	0.74	0.021	0.92
HOMA	0.145	0.55	0.211	0.38	0.126	0.60	0.198	0.41
Triglyceride	-0.172	0.51	-0.292	0.25	-0.120	0.64	0.174	0.50
LDL	0.092	0.73	-0.493	0.05	-0.010	0.97	0.031	0.91
HDL	-0.043	0.87	0.258	0.33	0.234	0.38	-0.501	0.04^*^
Total Cholesterol	-0.028	0.91	-0.415	0.11	0.141	0.60	0.341	0.19
Kisspeptin	0.132	0.50	0.428	0.02^*^			-0.110	0.57

**Figure 1:**
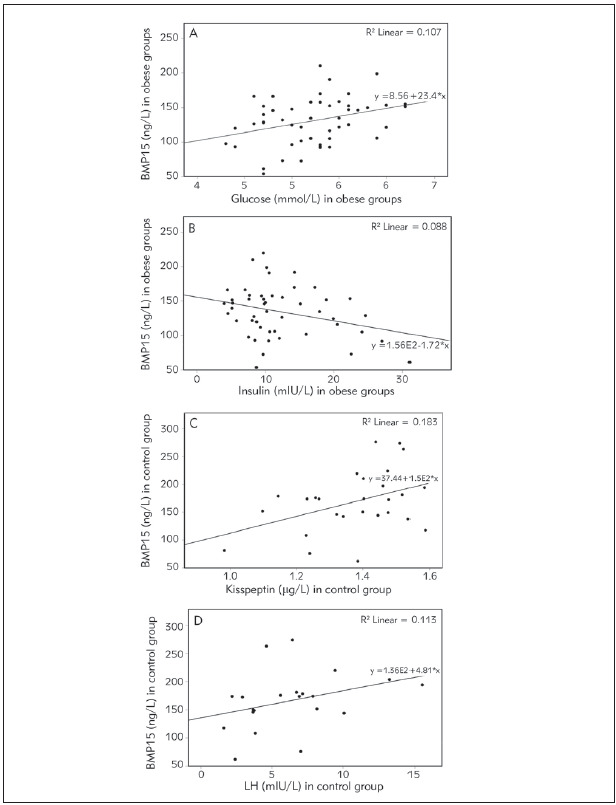
Correlation analyzes. (A) Correlation between BMP15 and glucose in obese groups; (B) Correlation between BMP15and insulin in obese groups; (C) Correlation between BMP15 and kisspeptin in control group (D) Correlation between BMP15 and LH in control group.

In control and both obese groups, statistically significant correlations are presented in Figure 1A, Figure 1B, Figure 1C and Figure 1D.

Except for a statistically significant positive correlation being identified between the kisspeptin and BMP15 concentrations in the control group, no correlation was found between the kisspeptin concentration and any other factors of interest in any of the groups. Moreover, no correlation was detected between the AMH concentration and the BMP15, GDF9, and kisspeptin concentrations in any of the groups.

In terms of the correlation analysis performed in relation to all of the participants in the present study, a negative correlation was identified between the participants’ BMI and their BMP15 and kisspeptin concentrations (r = -0.280, p = 0.009 and r = -0.319, p = 0.01, respectively), while a positive correlation was noted between the participants’ age and their GDF9 and BMP15 concentrations and BMI (r = 0.233, p = 0.02; r = 0.286 p = 0.007; and r = 0.239, p = 0.02, respectively). A negative correlation was found between the participants’ BMP15 concentration and their triglyceride and LDL levels (r = -0.312, p=0.02 and r = -0.305, p = 0.03, respectively), while a positive correlation was observed between the participants’ BMP15 concentration and their HDL level (r = 0.295, p = 0.03). Moreover, a positive correlation was determined to exist between the participants’ BMI and their glucose and HOMA levels (r = 0.364, p = 0.002 and r = -0.325, p = 0.01, respectively), while a negative correlation was found between the participants’ BMI and their HDL level (r = -0.300, p = 0.02).

## Discussion

The results of the present study indicate that obesity causes a significant decrease in the serum BMP15 and kisspeptin concentrations in women of reproductive age. However, no effect was identified on the part of obesity in relation to the serum GDF9 concentration.

The oocyte-secreted factors BMP15 and GDF9 work in synergistic cooperation with each other, and they are both essential for ovarian function [10] [11] [17].

It has previously been determined that GDF9 and BMP15 double mutations impair in vitro oocytematuration, fertilization, cumulus expansion, and preimplantation embryogenesis in mice [17]. Moreover, it has been shown that GDF9 and BMP15 mutations are associated with primary ovarian insufficiency (POI) [11] [18] [19]. Prior studies have indicated that impairment in terms of BMP15 secretion, activity, or synergy with GDF9 may impair ovarian function and, therefore, female fertility [11] [18] [19]. It has also been suggested that BMP15 may be a marker of oocyte quality [19]. The studies mentioned above have revealed the significance of BMP15 and GDF9 gene and protein defects in relation to reproduction. In the present study, obesity was found to reduce the BMP15 concentrations in women of reproductive age, and the identified decrease may be one factor leading to reproductive dysfunction in obese women.

To the best of our knowledge, the present study represents the first time that the GDF9, BMP15 and kisspeptin serum concentrations in reproductive women have been studied by classifying the participants into obese groups. Prior studies have been conducted with regard to intrafollicular fluids, although the BMP15 and GDF9 serum concentrations have solely been studied in relation to IVF patients. For instance, in a study conducted among women undergoing IVF, no differences in the mean serum BMP15 and GDF9 concentrations were found between the ovarian unstimulated and stimulated participants [20]. In addition, low serum GDF9 concentrations are considered indicative of low oocyte numbers following IVF. However, it should be borne in mind that the respective sensitivities of the kits used to measure the BMP15 and GDF9 concentrations were 24 pg/mL and 26 pg/mL, and further, the kits had not been standardized internationally [20]. Another prior study examined the relationship between the serum GDF-9 and BMP-15 concentrations and follicular fluid. While no statistically significant correlation was found between the serum GDF9 concentration and the follicular fluid, A positive correlation was identified between the serum BMP15 concentration and the follicular fluid [21].

In the present study, the kisspeptin concentrations in women of reproductive age were also investigated. It has previously been reported that kisspeptin is essential for fertility due to stimulating GnRH neurons via its receptor (i.e., *Kiss1r*) and so influencing the reproductive axis. Furthermore, kisspeptin is known to regulate metabolism, body weight (BW), and glucose homeostasis [16] [22] [23]. In a study conducted using gene-trap *Kiss1r-knockout* (KO) mice, it was determined that hypogonadism and gonadal steroid deficiency in both male and female mice. Also, both control and high-fat diets detected increased BW and adiposity, reduced energy expenditure, increased leptin levels, and impaired glucose tolerance in female mice [16].

A study performed using whole-body *Kiss1r-*KO mice similarly revealed increased adiposity and obesity with glucose intolerance in female mice, and both sexes (female and male mice) were severely hypogonadal [23].

Recent evidence has indicated that in addition to regulating fertilization, kisspeptin plays a critical role in metabolism, energy expenditure, and glucose homeostasis [16] [23]. In light of the findings of studies by Tolson et al. [16], kisspeptin dysfunction may lead to obesity and cause a vicious circle with regard to fertility, although further studies are required to confirm this.

The present study found that obesity reduces serum kisspeptin concentrations in women of reproductive age. In the literature, very few studies have investigated the serum kisspeptin levels in obese women of reproductive age. Interestingly, in these prior studies, different results were found in terms of the kisspeptin serum levels. For instance, no difference was found in the serum kisspeptin levels between overweight and normal-weight females in one study [24], while another study determined that the kisspeptin levels decreased in obese females compared to the control group [25].

The results of the present study suggested that the BMP15 and kisspeptin concentrations may beassociated with both metabolism and obesity. Moreover, obesity may exert detrimental effects on reproduction by decreasing the serum BMP15 and kisspeptin concentrations. The observed reductions in these factors may represent a milestone in relation to reproductive dysfunction and metabolic abnormalities in obese women.

In this study, while no correlation was found between the BMP15 concentrations and the glucoseand insulin levels in the control group, a positive correlation was identified between the BMP15 concentrations and glucose levels, as well as a negative correlation between the BMP15 concentrations and the insulin levels, in both obese groups. Furthermore, a statistically significant negative correlation was found between the BMP15 concentrations and the triglyceride and LDL levels, as well as a positive correlation between the BMP15 concentrations and the HDL levels in all of the participants in the present study. This suggests that the BMP15 concentration is closely related to metabolism. However, additional studies involving obese patients are required to corroborate the present results.

This study found a positive correlation between the BMP15 and kisspeptin concentrations and the LH levels in the control group, which suggests that a relationship may exist between the BMP15 and kisspeptin concentrations and the regulation of hypothalamic hormones.

In a prior animal study, the gene expression levels of both BMP15 and GDF9 were increased whenthe oocytes were matured in a medium containing kisspeptin compared with a medium without kisspeptin [15]. The results of this study supported the positive correlation result between bmp15 and kisspeptin in the present study.

A correlation was found between the BMP15 concentrations and the LH levels in the control group in the present study, and the absence of such correlation in the obese groups may indicate that reproduction was better in the control group.

In the present study, while the GDF9 concentration exhibited a statistically significant positive correlation with age in the control and all-participant groups, the BMP15 concentration exhibited a positive correlation with age in the obese and all-participant groups. These results appear to contradict the current understanding of these factors since they are known to be oocyte-specific factors, and further, oocyte numbers are known to fall with age [20] [21]. However, similar reports can be found in the literature. For instance, Riepsamen et al. [20] reported that in women of reproductive age (20–40 years), the serum BMP15 and GDF9 concentrations did not appear to decrease noticeably with age. Furthermore, they detected elevated GDF9 concentrations in some postmenopausal women [20]. Although this phenomenon is difficult to interpret, Riepsamen et al. [20] and Arifin et al. [21] suggested these factors potentially have a non-ovarian source, such as the pituitary, adrenal, granulosa, or cumulus cells, which may explain their increased concentrations in the peripheral serum.

## Conclusion

To the best of our knowledge, the present study reveals the first time that the BMP15 and kisspeptin serum concentrations were lower in obese reproductive women compared with the control group. The BMP15 concentrations may vary with the amount of adipose tissue, and BMP15 may interact with metabolic parameters such as insulin, glucose and lipids. The present findings suggest that the serum BMP15 and kisspeptin concentrations can be used to predict the success of infertility treatment in obese patients. As the significance of these findings is not yet clear, additional studies are required.

The limitation of this study was that if the sample size was relatively larger, the statistical significance of the p-value would be even more precise.

## Dodatak

### Acknowledgement

This study was supported by the Scientific Research Projects Coordination Unit of Kirikkale University (Project number: 2019/050).

### Authors Contributions

FBA; study conception and design, performing laboratory analyzes, analysis and interpretation of data, manuscript writing. NS; study conception and design, recruitment of volunteers, blood collection and data about patients, interpretation of data and revisal of the manuscript.

All authors have read and approved the final manuscript.

### Conflict of interest statement

All the authors declare that they have no conflict of interest in this work.
